# On the Employment of Anæsthetics in Obstetric Medicine and Surgery

**Published:** 1863-11

**Authors:** Horatio R. Storer

**Affiliations:** Boston, Surgeon to the Pleasant Street Hospital for Women


### MISCELLANEOUS.

### ON THE EMPLOYMENT OF ANÆSTHETICS IN OBSTETRIC MEDICINE AND SURGERY.

BY HORATIO R. STORER, M. D., OF BOSTON, SURGEON TO THE PLEASANT STREET HOSPITAL FOR WOMEN.

In ordinary surgical practice it would be viewed as cruel, if not decidedly wrong, to perform an operation without the previous induction of anæsthesia. This, however, is as yet often considered unsafe, unnecessary or unadvisable in obstetric practice, and in midwifery especially its aid is in this region, as a general thing, still withheld. In behalf, therefore, of those whose sufferings in the imperfect or abnormal performance of their peculiar function are doubtless far more exquisite and agonizing than we as men can possibly realize, I would claim precisely the same propriety and the same necessity for the use of anæsthetics in obstetrics as is now acknowledged in other and general practice.

The subject is one with which I happen to have been brought into peculiarly close relations; for the past eight years, and by a large circle of medical friends, I have been often importuned to state my convictions regarding it. I am satisfied that there exist several important and very prevalent errors, and in speaking decidedly it will be from extended personal experience.

Various objections have been brought against the employment of anæsthetics, but it will be found that their use has been advanced by the very arguments relied upon by their opponents. Many of these being upon their very face absurd, I shall notice only those that are in any degree plausible.

It has been asserted—

1.—That anæsthetics are hazardous to life;

2.—That they have a tendency to develop immortalities, alike in operator and patient;

3.—That it is unnecessary to abrogate pain, a natural phnomenon;

4.—That to do so is controversial of Scripture; and

5.—That their use is liable to produce subsequent ill effects upon the immediate or remote health of the patient.

Of these objections, two apply to the general use of anæsthesia, and the last three more especially to its employment in midwifery; though the last of them all, that involving a subsequent deleterious influence, to a certain extent has a general bearing. As to the first of them, which, with the exception of the last, is really the only one deserving serious consideration, it will be noticed that the argument applies with different force to ether and chloroform, the two anæsthetics generally employed; and to these again, with still other degree, as they may be resorted to in midwifery or for the other purposes of obstetric medicine or surgery.

I shall return to these points, and now merely state in answer to, first, the general objection that anæsthetics are hazardous to life;

*a.*—That anæsthesia is no more hazardous than other measures acknowledged by the profession to be not merely justifiable, but absolutely necessary; and

*b,*—That its use is often less hazardous than its absence.

To the second objection no more weight attaches than as regards the use of any narcotic or stimulant.

To the third, which covers the use of anæsthetics in labor, we reply that pain is of itself an evil, and of itself depresses the vital powers; that delays are here always dangerous to the life of either mother or child; that a naturally painless labor is almost never seen, and that to shorten the average duration of labor is to annually save tens of thousands of lives now sacrified.

The fourth objection applies equally to the whole practice of obstetric medicine and surgery, and therefore though it could be logically disproved, it needs no further reply.

The last objection to which we have referred, is based upon a belief that the use of an anæsthetic renders the patient, in general practice, more liable to affections of the circulation or nervous system, and in labor predisposes her to post-partum hemorrhage, etc. There is no doubt of this liability when the agent is an improper one or unskilfully administered, and it is to the frequency of such instances that we may fairly attribute the prevalent opinion. On the other hand, I do not hesitate to assert that, under other circumstances, no such fear need be entertained. As far as regards the possible sequelæ of child-bed, it will be seen that anæsthetics, when properly exhibited, increase the force of the uterine contractions, and probably, also, the very uterine contractility, so that in such cases liability to post-partum hemorrhage, for instance, would be decidedly lessened; and in abnormal labor, where the uterus itself, for operative measures, is purposely put to sleep, rapid delivery would be hardly likely to occur, unless by design, allowing the uterus, therefore, sufficient time to awaken again, as it would be sure to do. Should, however, hemorrhage take place under these circumstances, it would probably have occurred without the anæsthetic—for this agent does not separate the placenta from the uterine wall, any more than it produces, as has been gravely asserted of it in more than one instance, an hydrocephalic or an encephalous fœtus.

On the other hand, the obstetric advantages of anæsthesia are decided—giving<br>

to the patient<br>

relief from pain and<br>

saving of her vital powers—<br>

and to the operator<br>

increased facilities for action from muscular relaxation,<br>

and absence of disturbing elements,<br>

emotional and intellectual.

The indications for its use in obstetrics are—general and special.

1.—It is useful for purposes of diagnosis—both in cases puerperal and non-puerperal. It stops spasmodic and reflex muscular action, as in the various forms of hysteria, subduing general convulsive disturbances, quieting the abdominal muscles where their movement, regular or irregular, would suggest those of a fœtus in utero, flattening the surface in so-called spurious pregnancy, straightening joints supposed anchylosed or otherwise diseased, checking the extreme tenesmus of vagina or rectum, by which prolapsus uteri, cystocele or rectocele are at times stimulated; and in other cases it prevents the involuntary shrinking from pain, and consequent almost involuntary muscular action, during a severe examination.

2.—It relieves pain, anxiety and restlessness during
disease, as dysmenorrhœa, carcinoma, etc.;
operations, non-puerperal and puerperal;
and especially during labor—
thereby shortening it and lessening its mortality and dangers, to mother and child.

3.—It is indicated in labor, not merely because

*a.* it relieves pain, anxiety and restlessness, and so saves the vital powers, as already said; but because

*b.* it dilates the os and vaginal passage—often relieving rigidity where such exists;

*c.* it relaxes the voluntary muscles, preternaturally excited by reflex action, preventing their interference and undue effect;

*d.* it excites the uterine fibres, producing greater uterine contraction and thereby preventing inertia and hemorrhage;

*e.* it prevents puerperal convulsions where threatened, and where they are present it abates them;

*f.* it facilitates manual or instrumental assistance where such is required.

As to the relative value of the two anæsthetics for obstetric purposes: Between ether and chloroform, putting aside all local prejudices, which both in Europe and America have been allowed altogether too much weight, there are certain differences noticed, worthy of grave consideration. That I may not be misunderstood, I shall express myself very plainly, and in view of the circumstances under which I have experimented with each of these agents,* I trust the profession will feel inclined to look fairly at my views of the subject, even if in some respects they run counter to the generally received opinion.

I think I may state the following as rules for practice:

1.—Ether alone, and never chloroform, should be used for purposes of diagnosis and in all cases of operative surgery, capital or minor, general or obstetric, except those immediately pertaining to labor.

* My first impressions and estimate of ether were formed in Boston, from direct observance of its effects in the hands of those who first applied it to practice, and who have ever since kept its best interests in view. I refer to these sources in connection with my own private experience with the agent, now by no means inconsiderable, inasmuch as they have all led me to a single conclusion. My first impressions and estimate of chloroform, against which I had been decidedly prejudiced, were formed from daily, I might say hourly, familiarity with it during my sojourn in Edinburgh with Prof. Simpson, who, while he was the first ever to use ether in midwifery, was only led to discover the anæsthetic properties of chloroform, at deliberate and repeated risk to his own life, by experience of the disadvantages of ether for the purposes of labor.

2.—Chloroform alone should be used in midwifery, to the entire exclusion of ether.

That deaths have taken place in general practice from the use of chloroform, I freely admit. It is remarkable, however, that many of these cases have been of the simplest operations, as in dentistry, and that death often occurred before the operation had commenced, the agent having been exhibited not to lessen but to prevent pain, the nervous system being in a quiescent condition.

For the ordinary purposes of surgery, therefore, it is plain that as less risk in such cases does pertain to ether, it should be used in preference to chloroform. With regard to the practice of midwifery, however, it is far different. To the present date, so far as I am aware, there does not exist on record, from the thousands of obstetric cases in which chloroform has been used, a single instance where death can be legitimately attributed to its influence. With certain allegations to the contrary I am of course familiar, but in the cases upon which these are based, the fatal result seems in every instance to have been directly dependent, not upon chloroform, but upon one or other of the following causes:

The agent was impure, or
was administered by an incompetent attendant,
whether whysician or nurse;
the patient, without other care or supervision, herself induced the anæsthesia,
either during the labor
or subsequently—or
there existed some previous disease or unavoidable complication, that of itself must necessarily have produced death.

Such being the fact, the objection falls. It cannot be said that if not on record, unfortunate cases, directly depending upon chloroform, must yet have occurred; for there are too many opponents of anæsthesia, who would at once seize upon and publish them did they exist.

If such immunity in childbed be granted to chloroform, as I conceive must be done, upon what grounds can it be explained? Upon several.

Firstly: labor, though so often treated and spoken of to the contrary, is essentially a normal and strictly physiological action—the great end for which, sexually and anatomically speaking, woman was formed. The shock, therefore, to the system which she undergoes during child-bed, though in the simplest cases so tremendous, is one for which, to a great extent, provision has already been made. There is at that time a greater tolerance of nervous shock, for want of a better expression, than we find in ordinary surgical cases of apparently much less proportionate severity, especially if these be in disease of long standing, or after severe accident, where the vital powers have been in consequence undermined, or an important organ has been structurally disorganized. In these cases the vitality of the patient may be considered as below par; in labor, on the contrary, it is decidedly exalted, and above par.

Upon this point, the obstetric tolerance of chloroform, other elements seem to bear, as

Secondly: the excitability of the reflex system in the female is notorious; and that this is enhanced not merely by abnormal processes, as of various uterine or ovarian disease, but even by the perfectly healthy performance of natural functions, as of menstruation, copulation and conception. This influence is very evident during the whole term of gestation, and it is undoubtedly as powerful during labor. If it were granted that the liability to fatal depression or collapse from the use of chloroform existed during parturition to so great a degree as at other times, against which, however, we have other reasoning and direct negative evidence besides, it is probable that in the very exaltation of the whole reflex system to which I refer, we have a sufficient safeguard and cure.

But still further:

Thirdly: It is now generally believed that in the female, during the period of menstruation, a large elimination of carbon from the sanguineous system takes place through the medium of tho uterus, and that at these times, accordingly, the lungs are relieved of a portion of their usual work. If this be true, and there is certainly strong evidence in its favor, then it follows, normal labor taking place almost precisely at the time of the periodical menstrual molimen,* that a certain amount of adverse impression might be produced at this time upon the general system through the lungs, which could not safely be induced by the same channel at another.

By the three theories I have now propounded, namely, (1) the gradual preparation of the system for the shock of parturition, (2) the existence of an unusual, and for the time tonic, stimulus to the nervous system, by which cardiac paralysis may be averted, and (3) an unusual, and for the time tonic, depuration and decarbonization of the blood through the ute-

* This molimen undoubtedly occurs to a certain extent, though perhaps almost imperceptibly, at its regular interval throughout gestation, rendering the patient much more liable to abort at some times than others upon slight provocation.

rine sinuses, by which the ordinary tendency to asphyxia from the use of chloroform may be prevented—do we not have a satisfactory explanation of the immunity from accident that has been observed in the exhibition of this agent during childbirth?*

I have dwelt at length upon this point in my brief summary, the immunity of chloroform during labor, because its apparent inexplicability has been to many a sufficient reason to decide them at once against its use. "We grant that a death may never yet have occurred from chlorofor n in childbed," has more than once been said to me by friends of high authority, "but you may possibly lose your next patient, and are therefore not justified in such hazard." I confess that early in practice I shared these fears, but since the arguments now urged have suggested themselves, such scruples have gone, and of late I have not hesitated to administer chloroform to parturient patients far gone in cardiac and pulmonary disease.

The arguments above advanced have not, I think, been hitherto as distinctly presented by any writer or teacher, though in part they may have been foreshadowed.† Do they not explain certain other intricate obstetric problems? As, for instance, the alleged improvement of phthisical women during pregnancy; the apparent relief to pulmonary disease sometimes seen, when complicated with amenorrhœa, during vicarious menstruation; and also the rapid decline in consumptive patients, occasionally occurring after parturition. I would call the attention of thoracists to these several points.

To return—

The use of chloroform in midwifery, granting, as I have claimed, its safety for this purpose, has certain positive advantages over ether; sufficient, I consider, to entitle it to decided preference.‡

* It might be thought that the last of the theories proposed would apply with equal force to the case of purely venous hemorrhage from any ordinary source. I conceive, however, that even were we to allow a certain amount of influence in such cases, which have not as yet in this connection been at all investigated, it is the fact of the occurrence as a regular and normal physiological phenomenon during labor, no matter how small in extent, that furnishes the key to the whole question.

† I frankly acknowledge that my attention was first riveted upon this question some thirteen years ago by my friend Dr. Walter Channing, to whose philosophical remarks upon the subject in his excellent treatise upon Etherization in Childbirth, I would refer my readers.

‡ To these I called the attention of the profession several years since, at a meeting of the Suffolk District Medical Society, at which it had been proposed that the physicians of this city should once for all stamp their emphatic and general condemnation upon the inhalation of chloroform. I then claimed that whatever objections might be urged against the drug for ordinary practice, an exception must be made in its favor for cases of midwifery, promising that at a future day I would revert to the subject. I accordingly now redeem this pledge.

1.—The vapor of chloroform is much more agreeable to the patient and to the physician.

2.—It is less likely to occasion any unpleasant or depressing concomitant, as nausea, vomiting, etc.

3.—Being more powerful than ether, it induces anæsthesia with much more rapidity—a matter of great importance in labor, where it is always necessary, except where operative interference is required, that the effect of the anæsthetic should be confined to the pains, and not pass over into the interval,

4.—Its effects are much more transient than those of ether, a characteristic of equal value with the last, and for precisely the same reason, namely, that

5.—It does not, as is frequently the case, with ether,* prevent the recurrence of the pains, and so stop the progress of the labor.

6.—It is more efficacious than ether for restraining or preventing puerperal convulsions and puerperal mania.

It has been suggested to me by a close observer, Dr. McIntire, now of Concord, N. H., whose use of chloroform in childbed has been very extensive and dates from its first suggestion to the profession, that when resorted to there is much less danger of puerperal fever, if the patient, as is often the case, has been directly exposed to contagion or any other exciting cause. From the facts communicated to me by Dr. McIntire, I am inclined to think there are good grounds for his opinion. There is no doubt, at any rate, of the efficacy of chloroform in preventing exhaustion, nervous irritation and other predisposing causes.

As to the time of its administration, a point upon which there has been much difference of opinion:

Generally, its use is hardly required till the completion of the first stage of labor, when the os uteri has become fairly dilated. Should there exist, however, sufficient suffering at an earlier period, the agent should certainly then be resorted to. It should be given only during the pains, except a complication exist requiring manual or instrumental interference, when its use should be continued through the interval; and in this lies one of the chief advantages of chloroform in midwifery, that whereas given during the pains alone, and properly, it not only does not interfere with the uterine

* The liability of ether in this respect is notorious. For a single admission to the point, and among many that might be adduced, I will refer to editorial articles in the *Boston Med. and Surg. Journal* for August of the present year, (pp. 63 and 87,) published *after* the above paper was publicly read.

contractions, but regulates, if inconstant, and enhances them, on the other hand, if a cessation of that action be required to enable us safely to pursue any measures within the cavity of the uterus, as for turning or applying forceps above the brim, we can obtain it by extending the use of the agent through the interval. In a large proportion of cases it will not be necessary, at any time during the labor, to induce complete insensibility; a very few breaths of chloroform, sometimes indeed a single one, sufficing to annul the sensation of pain.

The absolute amount given is usually too small and with too sparing a hand. Somewhat like opium, we get from minute doses a period of excitement and perhaps of delirium that is escaped by more decided application. The great secret is to produce the narcotism as rapidly as possible, and yet gradually obtain our mastery over the respiratory organs. This remark applies with equal force to the administration of ether in ordinary surgical practice, though its importance is too often lost sight of or not fully appreciated.

At first, and throughout, atmospheric air should be freely admitted with the vapor applied; and therefore I would condemn any form of artificial inhaler, however carefully oonstructed. The simplest form is the best, and a mere handkerchief or napkin will answer every indication if it be only borne in mind that the vapor of chloroform is much heavier than air, and if properly applied will descend about the face of its own weight.* Attention to this fact will also prevent the possibility of vesicating or unduly irritating the mucous or cutaneous surfaces. The patient should be told from the outset to inspire very deeply; the motion soon becomes automatic, and the vapor, by penetrating every pulmonary vesicle, produces a much more profound and instantaneous effect. Throughout the inhalation and as a matter of course, due attention should be given to the pulse, and more especially to the respiration of the patient.

I have referred to the necessity of the agent being perfectly pure and reliable. In this matter perhaps I may be over-cautious; but upon personally inhaling many specimens of chloroform, procured from different

* A suggestion has been made to me by Dr. Sutherland, the well-known Professor of Chemistry at Montreal, that may prove of extreme value in preventing the occurrence of accident from chloroform in ordinary surgical practice. It is that the face and body of the patient during inhalation should be turned more to one side than is generally the case. The weight of the vapor being such as after a few inspirations to fill and almost hermetically seal the lungs by its mere gravity, the position I have indicated would evidently allow more perfect expiration and a much more complete entrance and admixture of atmospheric air than is otherwise possible.

sources, there has apparently been evident a diversity of effect, and I therefore still confine myself to what from long experience I have every reason to be satisfied with—the manufacture of Messrs. Duncan & Flockhart, of Edinburgh, procuring it either through friends or responsible parties in the trade.*

Of chloric ether I have had much less experience than of sulphuric ether and chloroform; knowing no reason to prefer it to either of these agents, while there are several decided objections to its use, I omit its further mention.

It is sometimes asked, if a patient should be urged to the use of an anæsthetic, when timid or prejudiced against it. This is a question that, personally, I have no hesitation in answering affirmatively. These fears, as already said, are perfectly groundless, when the agent is properly given and its use duly restricted. The risk to life in labor lie rather in the absence of an anæsthetic than in its administration, and so does the liability to a tedious recovery. Few, if any patients, and this remark applies also to cases of general surgery, but can safely bear an anæsthetic, and come kindly under its influence, too, if it be properly exhibited; and every additional example of this that we may be able to present in practice, is so far a refutation of the belief to the contrary that so generally obtains. For this reason I should advise its use under the circumstances we are now considering, but not for this alone. Since entering obstetrical practice, it has been with me a matter of conscience, this abolishing the last and most exquisite agony of all, save dissolution, to which, in one respect, the rending asunder of two distinct natures during childbirth, it bears no slight resemblance.

I can recall not one single case of labor among several hundred where I have given chloroform, in which, however simple or complicated the case, I have noticed the slightest ill effect from the anæsthetic; in all, I am satisfied, its use was attended with benefit to the patient. I refer to this per-

* Messrs. Metcalf & Co. and Leopold Babo, of Boston, are prepared, I believe, to furnish chloroform directly from Messrs. Duncan & Flockhart.

The above rules one would suppose to be simple enough. With reference to the objection made, as in this Journal for October 15th, page 228, that ignorance of these plain and reliable formulæ as to the administration of chloroform, because, common, is sufficient argument against learning them, it applies equally to every drug of any power used by medical men. Because accidents have happened, in the hands of the ignorant, from their exhibition in surgery, the agent is not to be blamed or lightly thrown aside; that accidents have happened from their exhibitions in the hands of the wise and skillful, who were yet on an important point or points ignorant, careless or forgetful, should no more be laid to the agent's discredlt.

sonal experience for the same reason that has controlled my practice—that I believe that in the advancement of medicine, individual influence but *begins* with the cases, be they few or many, under a physician's care. It is the example and the embodied thought that avail.

